# Wogonin upregulates SOCS3 to alleviate the injury in Diabetic Nephropathy by inhibiting TLR4-mediated JAK/STAT/AIM2 signaling pathway

**DOI:** 10.1186/s10020-024-00845-4

**Published:** 2024-06-06

**Authors:** Yufeng Liu, Mengbi Zhang, Lu Zeng, Yanhong Lai, Songzhao Wu, Xiaoyan Su

**Affiliations:** 1 Nephropathy Department, DongGuan Tungwah Hospital, Dongcheng, East Road No. 1, DongGuan, 523015 Guangdong China; 2 Dongguan Key Laboratory of Precise Prevention & Treatment of Chronic Kidney Disease and Complications, DongGuan, 523015 Guangdong China

**Keywords:** Wogonin, SOCS3, TLR4, Diabetic nephropathy, JAK/STAT signaling pathway, Inflammasome

## Abstract

**Background:**

Diabetic nephropathy (DN) is a life-threatening renal disease and needs urgent therapies. Wogonin is renoprotective in DN. This study aimed to explore the mechanism of how wogonin regulated high glucose (HG)-induced renal cell injury.

**Methods:**

Diabetic mice (db/db), control db/m mice, and normal glucose (NG)- or HG-treated human tubule epithelial cells (HK-2) were used to evaluate the levels of suppressor of cytokine signaling 3 (SOCS3), Toll-like receptor 4 (TLR4), inflammation and fibrosis. Lentivirus was used to regulate SOCS3 and TLR4 expressions. After oral gavage of wogonin (10 mg/kg) or vehicle in db/db mice, histological morphologies, blood glucose, urinary protein, serum creatinine values (Scr), blood urea nitrogen (BUN), superoxide dismutase (SOD), glutathione (GSH), and reactive oxygen species (ROS) were assessed. RT-qPCR and Western blot evaluated inflammation and fibrosis-related molecules.

**Results:**

HG exposure induced high blood glucose, severe renal injuries, high serumal Src and BUN, low SOD and GSH, and increased ROS. HG downregulated SOCS3 but upregulated TLR4 and JAK/STAT, fibrosis, and inflammasome-related proteins. Wogonin alleviated HG-induced renal injuries by decreasing cytokines, ROS, Src, and MDA and increasing SOD and GSH. Meanwhile, wogonin upregulated SOCS3 and downregulated TLR4 under HG conditions. Wogonin-induced SOCS3 overexpression directly decreased TLR4 levels and attenuated JAK/STAT signaling pathway-related inflammation and fibrosis, but SOCS3 knockdown significantly antagonized the protective effects of wogonin. However, TLR4 knockdown diminished SOCS3 knockdown-induced renal injuries.

**Conclusion:**

Wogonin attenuates renal inflammation and fibrosis by upregulating SOCS3 to inhibit TLR4 and JAK/STAT pathway.

**Supplementary Information:**

The online version contains supplementary material available at 10.1186/s10020-024-00845-4.

## Introduction

Diabetic nephropathy (DN) contributed to about 40% of diabetes mellitus-related renal diseases (Zoja et al. 2020). Its characteristics include persistent albuminuria, gradually decreased glomerular filtration rate (GFR), and increased arterial blood pressure (Sagoo and Gnudi 2020). At the molecular level, HG-induced hyperglycemia and urinal proteins are major predisposition factors for DN (Wolf 2004). Recently, increasing evidence has shown that many factors, including increased inflammation and the activation of the renin-angiotensin system, are attributed to DN (Wolf 2004). Regarding the complex pathobiology of the diabetic kidney, the treatment of DN is very challenging. Thus, it is very urgent to explore the molecular mechanism underlying the pathological development of DN. Wogonin from the traditional Chinese herb Georgi inhibits HG-induced vascular inflammation, indicating that wogonin is a promising candidate for treating diabetic vascular diseases (Ku and Bae 2015).

Recently, several studies showed that wogonin exhibited protective roles in DN via regulating inflammatory response, cell apoptosis, oxidative stress, and fibrosis (Lei et al. 2021; Liu et al. 2022; Zheng et al. 2020), suggesting that wogonin exhibited therapeutic potential for diabetic renal diseases. However, few reports are related to the function and potential mechanism of diabetic nephropathy.

Toll-like receptors (TLRs) are one of the significant receptors responsible for diabetes mellitus-related inflammatory response. The previous study showed that high glucose activates TLR and leads to an inflammatory response in diabetic diseases, such as diabetic retinopathy (Liu et al. 2020; Zhu et al. 2017) and diabetic nephropathy (Zhang et al. 2020). TLR4 participated in HG-induced inflammation, possibly by regulating NF-Κb-mediated cytokine secretion (S. Zhang et al. 2020a, b). The previous experiment indicated that high glucose activated the JAK/STAT signaling pathway to contribute to diabetic vascular dysfunction (Zhou et al. 2019). The additional experiment also showed that high glucose activated the renin-angiotensin system (RAS), which contributed to the development of DN by the increased oxidative stress (Leite et al. 2019). Accumulated studies indicated that the dysregulation of TLR4 signaling and RAS activation were essential for DN’s development. Therefore, it is valuable to explore the mechanism related to their potential cross-regulation during the development of DN to find a new therapeutic target.

The suppressor of cytokine signaling 3 (SOCS3) can negatively regulate JAK and STAT3 activation (Yoshimura et al. 2005). High glucose led to hypermethylation of SOCS3 promoter via DNA methyltransferase 1 (DNMT1). Thus, it decreases SOCS3 expression; the downregulated SOCS3 facilitates STAT3 activation, contributing to diabetic fibrosis (Tao et al. 2021). A previous study showed that wogonin-induced SOCS3 expression after STAT3 was blocked (Nam et al. 2014). Under HG exposure, SOCS3, p-JAK2, and p-SAT3 were highly upregulated, indicating that SOCS3/JAK2/STAT3 axis plays a vital role in hyperglycemia-induced epithelial cell injury (Duan et al. 2017). SOCS3 suppresses inflammatory activity by mediating TLR4-dependent inflammasome activation(Zhang et al. 2020a, b). During the development of diabetic diseases, high glucose remarkably activated the NLRP3 inflammasome via upregulating MAPK activation (Zhang et al. 2020a, b). However, there is still no report about whether SOCS3 contributes to HG-induced inflammasome activation.

In the preliminary experiment, we found that SOCS3 was downregulated and TLR4 was upregulated under diabetic conditions. Based on the above experiments, we aimed to explore the effects of wogonin on HG-induced DN. Using in vitro and in vivo diabetic models, we further investigated whether wogonin ameliorated DN by regulating SOCS3 to decrease TLR4-dependent inflammatory response.

## Materials and methods

### Animals and experimental design

Age-matched C57BLKS/J db/db (week 10, male, *N* = 40) and db/m mice (week 10, male, *N* = 16) were purchased from Beijing Huafukang Biotechnology Co., Ltd. The Committee Ethics approved all animal experiments at Dongguan Tungwah Hospital. All mice were housed in the University animal facility with a 12 h light/dark cycle and free to access water and pellet chow. Diabetic mice were randomly divided into four groups (*n* = 8 for each group): db/db, db/db + wogonin, db/db + wogonin + sh-SOCS3, db/db + wogonin + sh-SOCS3 + sh-TLR4. Db/m mice were used as the normal control. Mice in diabetic groups were administered 40 mg/kg wogonin (W0769, Sigma-Aldrich, MA, USA) every other day for 12 weeks (Liu et al. 2022; Zheng et al. 2020). Control mice (db/db) were given the same volume of dimethyl sulfoxide (DMSO, 0.1 mL). To inhibit SOCS3 and TLR4 genes, we injected the diabetic mice intravenously with 50 µL of lentivirus carrying SOCS3, TLR4, or negative control with a 1 × 10^11^ virus particles/mL titer. The mice in the db/db + wogonin group were injected with the same volume of saline as a control. Mice were sacrificed at week 22. Subsequently, we collected the kidney, blood, and urine for subsequent analysis.

### Blood glucose and insulin level

To confirm the successful establishment of diabetic mice, we performed intraperitoneal glucose tolerance test (IPGTT) and intraperitoneal insulin tolerance test (IPITT) after 12-week feedings. After fasting for 6 h, glucose (2.0 g/kg body weight) or insulin (0.75 U/kg body weight) was intraperitoneally administered. After the injection (0, 15, 30, 60, and 120 min), the blood from the tail vein was collected to measure the glucose and insulin levels using a Freestyle blood glucose monitoring system in NJ, USA.

To measure blood glucose, we collected the blood from the tail vein of mice after overnight fasting at the indicated time points (days 6–22). Glucose levels were detected using a glucose colorimetric kit (EIAGLUC, Thermo Fisher Scientific, MA, USA).

### Biochemical assays

To measure blood urea nitrogen (BUN), serum creatinine values (Scr), alanine aminotransferase (ALT), aspartate aminotransferase (AST), and alkaline phosphatase (ALP), mice performed cardiac punctures to collect the blood (200–300 µL). The corresponding commercial kits measured the BUN, Scr, ALT, AST, ALP (EIABUN, Thermo Fisher Scientific, MA, USA, and SKT-217-192, Eagle Biosciences, NH, USA) with detailed instructions. For the urine albumin, we measured 24-hour urinary albumin excretion levels using a mouse albumin ELISA kit (E99-134, Fortis Life Sciences, MA, USA).

### Hematoxylin and eosin (H&E) and periodic acid-Schiff (PAS) staining

After the mice were sacrificed, the kidneys were harvested and fixed with 4% paraformaldehyde (PFA). The sections were conducted into 10 μm thickness. Subsequently, hematoxylin and eosin were used to analyze the pathological structure changes (H&E, H-3502-NB, Novus Biologicals, LLC, CO, USA). The glycogen levels in tissues were evaluated using a periodic acid-Schiff (PAS) stain kit (AR165, Agilent, CA, USA). The distributions of glycosylated proteins were observed under a light microscope (DP-71; Olympus, Tokyo, Japan).

### Cell culture and high glucose administration

Human renal proximal tubule HK-2 cells (CRL-2190™, ATCC, VA, USA) were maintained in keratinocyte serum-free medium (K-SFM, 17005-042, ATCC, VA, USA) in a cell culture incubator. HK-2 cells were subject to 30 mmol/L of glucose for 24 h as high glucose exposure(Cui et al. 2023; Li et al. 2021; Liu et al. 2023). To investigate the roles of wogonin in HG-induced cell toxicity, we exposed HK-2 cells to increasing concentrations of wogonin (1, 2, 4, 8, 16, 32, and 64 µM) for 24 h to determine the suitable concentration for acute toxicity. We chose 8 µM as an optimal concentration for the co-treatment with wogonin and HG based on the cell viability. The treatment with mannitol (8 µM) for 24 h was used as the negative control. Mannitol plus HG medium was used as a control. In brief, cells were divided into three groups: (1) negative control, (2) HG, and (3) HG + wogonin. For in vitro cell viability, the assays were conducted using a commercial CCK-8 kit (96,992, Sigma-Aldrich, Beijing, China).

### Cell transfection

We conducted loss- and gain-of-function assays using lentivirus infection to evaluate the role of SOCS3 and TLR4 in vitro and in vivo. We purchased lentivirus expressing shRNA- or ov-SOCS3 or TLR4. After 24 h, the spent medium was changed into the complete HK-2 medium containing HG (30 mM) or HG (30 mM) + wogonin (8 µM). After a further 48 h, the cells were collected for future assays.

### RT-qPCR

After stimulation, total RNAs were extracted using an RNeasy mini kit (Qiagen, CA, USA). 1.0 µg of total RNA was reversely transcribed into cDNA using a commercial kit (4,387,406, Thermo Fisher Scientific, MA, USA). RT-qPCR was conducted using SYBR™ Green One-Step RT-qPCR Kit (11,736,059, Thermo Fisher Scientific, MA, USA ). The used primers are listed below: human SOCS3 F, 5- CCTGCGCCTCAAGACCTTC-3; R, 5- GTCACTGCGCTCCAGTAGAA-3; human TLR4 F, 5- CCTCGGCGGCAACTTCATAA-3; R, 5- AGAGCGGATCTGGTTGTACTG-3; human β -actin F, 5- CCCTGGAGAAGAGCTACGAG − 3; β-actin R, 5- CGTACAGGTCTTTGCGGATG − 3. Mouse SOCS3 F, 5- GCGGGCACCTTTCTTATCC-3; R, 5- CTGGAGGCGGCATGTAGTG-3; mouse TLR4 F, 5- ACCTGGCTGGTTTACACGTC − 3; R, 5- CTGCCAGAGACATTGCAGAA − 3; mouse β-actin F, 5- ATATCGCTGCGCTGGTCG − 3; β-actin R, 5-CCTTCTGACCCATTCCCACC-3. The corresponding sample evaluated the relative quantifications of SOCS3 and TLR4 by the 2^−△△ Ct^ method. β-actin was used as an internal control.

### Measurement of ROS, MDA, GSH, and SOD content

Cells were stained by fluorescent dye 2′,7′-dichlorodihydrofluorescein diacetate (H2DCFDA, D399, Thermo Fisher Scientific, MA, USA) to detect intracellular reactive oxygen species (ROS). A Nikon A1R laser scanning confocal microscope (Nikon Corp., Tokyo, Japan) was used to image cells after staining.

After treatment with vehicle, HG, or HG + wogonin, the corresponding commercial kit was used to detect the levels of malondialdehyde (MDA, NWK-MDA01), glutathione (GSH, NWK-GSH01), superoxide dismutase (SOD, NWK-SOD02) in HK-2 cells of each group according to the detailed instructions (Northwest Life Science Specialties, LLC, WA, USA).

### ELISA assay

To assess the cytokine secretion after stimulations, we purchased the respective commercial ELISA kit for TNF-α, IL-1β, IL-6, IL-8, circulating adiponectin, and leptin from Biolegend, CA, USA. Albuminuria and urinary albumin-to-creatinine ratio (ACR) were detected using Mouse Albumin ELISA Quantification Set (E99-134, Fortis Life Sciences, MA, USA) and Creatinine Companion kit (EIACUN, Thermo Fisher Scientific, MA, USA), respectively. The absorbance was detected at 450 nm using a 96-microplate reader (GM3000, Promega, WI, USA). The quantification of cytokines was calculated using a standard curve.

### Western blot

After collection, HK-2 cells and renal tissues were lysed using RIPA buffer (89,900, Thermo Fisher Scientific, MA, USA) containing 0.1 M phenylmethylsulphonyl fluoride (PMSF, 36,978, Thermo Fisher Scientific, MA, USA). The supernatant was collected after pre-clear by centrifugation (12,000 g × 15 min) at 4℃. Protein concentration was determined using a BCA protein assay kit (23,227, Thermo Fisher Scientific, MA, USA). 20 µg of total proteins for each sample were subjected to 12% SDS-PAGE and transferred to Nylon membranes (LC2003, Thermo Fisher Scientific, MA, USA). After blocking with 5% non-fat milk in 1 × Tris-buffered saline with 0.1% Tween® 20 Detergent (TBST), the membranes were incubated with the corresponding primary antibodies (shown in Table 1). After three washes by 1×TBST, the membranes were incubated with the corresponding horseradish peroxidase-labeled secondary antibody (shown in Table 1). The membrane was developed using an ECL substrate kit (ab65623, Abcam, MA, USA). The protein bands were analyzed by Image Lab Software 6.1 (Bio-Rad, CA, USA).

**Table 1:** List of antibodies used in this study

**Primary antibodies**			
Target proteins	Host	Catalogue No.	Sources
SOCS3	rabbit	ab280889	Abcam, MA, USA
TLR4	rabbit	19811-1-AP	Proteintech
collagen 1	rabbit	ab26004	Abcam, MA, USA
connective tissue growth factor (CTGF)	rabbit	ab209780	Abcam, MA, USA
fibronectin (FN)	rabbit	ab2413	Abcam, MA, USA
TGF-β1	rabbit	ab179695	Abcam, MA, USA
angiotensin-converting enzyme 1 (ACE)	rabbit	ab254222	Abcam, MA, USA
Angiotensin II (Ang II)	mouse	MA1-82996	Thermo Fisher Scientific, MA, USA
angiotensin II type 1 receptor (AT1R)	rabbit	ab124734	Abcam, MA, USA
p-JAK2	rabbit	ab32101	Abcam, MA, USA
JAK2	rabbit	ab108596	Abcam, MA, USA
p-STAT1	rabbit	ab109461	Abcam, MA, USA
STAT1	rabbit	ab109320	Abcam, MA, USA
p-STAT3	rabbit	ab76315	Abcam, MA, USA
STAT3	rabbit	ab68153	Abcam, MA, USA
AIM2 (AIM2)	rabbit	ab119791	Abcam, MA, USA
ASC	rabbit	ab150368	Abcam, MA, USA
caspase-1	rabbit	ab207802	Abcam, MA, USA
IL-18	rabbit	ab243091	Abcam, MA, USA
IL-1β	rabbit	ab315084	Abcam, MA, USA
GAPDH	rabbit	ab9485	Abcam, MA, USA
**Secondary antibodies**			
Anti-Rabbit IgG H&L (HRP)	Goat	ab205718	Abcam, MA, USA
Anti-Mouse IgG H&L (HRP)	Goat	ab205719	Abcam, MA, USA

### Immunoprecipitation (IP assay)

HK-2 cells were transfected with pCMV empty vector or pCMV SOCS-3 for 48 h. Then, cells were lysed using IP Lysis/Wash Buffer supplemented with Halt™ Protease (Product No. 78,430) and Phosphatase (Product No. 78,428) Inhibitor Cocktails. Subsequently, Co-Immunoprecipitation was performed using a commercial Pierce™ Co-Immunoprecipitation Kit (PI26149, Thermo Scientific™, MA, USA) according to the detailed manual. The rabbit anti-SOCS3 (ab280884, Abcam, MA, USA) and rabbit anti-TLR4 (19811-1-AP, Proteintech, CA, USA) were used individually to detect the expressions of SOCS3 and TLR4.

### Statistical analysis

All data were represented as Mean ± standard deviation (SD). All experiments were conducted three times. The statistical difference was analyzed using an unpaired two-tailed t-test or one-way ANOVA of variance with the post hoc Tukey test by GraphPad Prism 9.0 (CA, USA). *P* < 0.05 was considered statistical significance.

## Results

### SOCS3 and TLR4 expression in the diabetic and control renal tissues

**Fig. 1:**
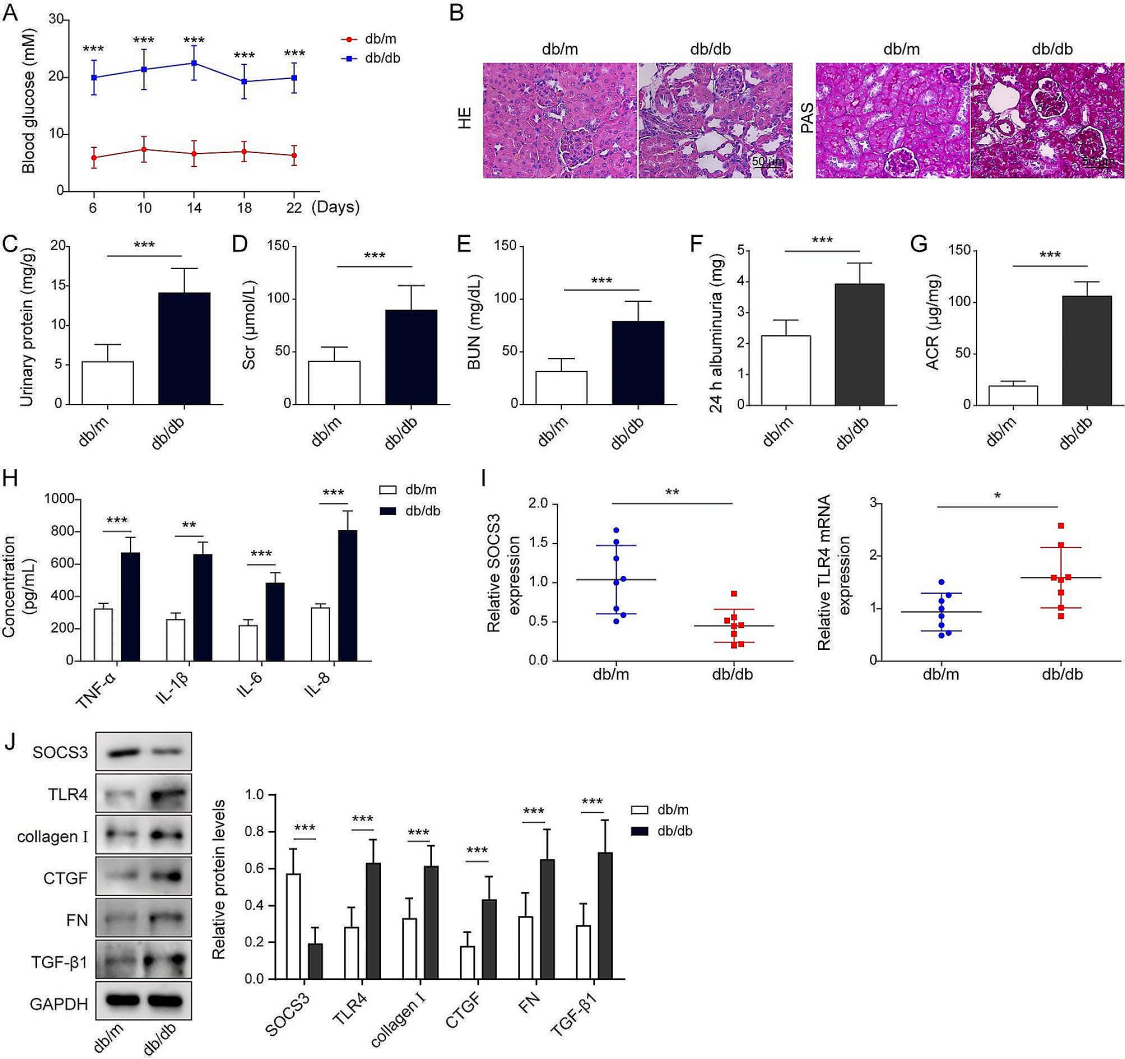
SOCS3 and TLR4 expression in the diabetic and control renal tissues. (**A**). Blood glucose levels were detected in tail vein blood. (**B**). Histopathologically examine the renal tissues using hematoxylin, eosin (HE), and periodic acid-Schiff base (PAS) staining. Scale bar = 50 μm. (**C**-**E**). An automatic biochemical analyzer measured the content of urinary protein, serum creatinine values (Scr), and blood urea nitrogen (BUN), respectively. (**F**-**G**) 24-hour albumin and urinary albumin-to-creatinine ratio (ACR) were detected by ELISA assay. (**H**). ELISA assay detected the contents of cytokines in mice sera. (**I**). SOCS3 and TLR4 expressions were detected by RT-qPCR in renal tissue. (**J**). Western blot measured the protein levels of SOCS3, TLR4, collagen I, CTGF, FN, and TGF-β1 in renal tissues. Data are representative of three independent experiments. *P < 0.05, **P < 0.01, ***P < 0.001 versus the corresponding control

As shown in Fig. 1A, the db/db mice group exhibited significantly higher blood glucose levels than those in the db/m group. The H&E and PAS staining showed those db/db mice exhibited obvious renal tubular and interstitial fibrosis, mesangial matrix hyperplasia, and increased thickness of basement membrane (Fig. 1B). Further experiments indicated that db/db mice showed an increased level of urine albumin related to that in db/m mice, suggesting the dysfunction of the kidney (Fig. 1C). Both of serum creatinine (Scr) and blood urea nitrogen (BUN) were also remarkably increased in db/db mice relative to db/m mice (Fig. 1D-E). We also found that db/db mice exhibited higher levels of 24-hour albumin and urinary albumin-to-creatinine ratio (ACR) (Fig. 1F-G). Meanwhile, the levels of cytokines, including TNF-α, IL-6, IL-1β, and IL-8, were dramatically upregulated in db/db mice related to those of db/m mice (Fig. 1H). But, SOSC3 expression was decreased, and TLR4 expression was increased in db/db mice compared with db/m mice **(**Fig. 1I-J). Similarly, renal fibrosis-related molecules collagen I, CTGF, FN, and TGF-β1 were also elevated. These findings indicated that diabetes exhibited decreased SOCS3 and increased TLR4, contributing to DN’s development **(**Fig. 1I-J). Additionally, experiments showed that the ov-SOCS3 vector increased SOCS3 levels relative to the ov-NC empty vector, but the sh-SOCS3 vector decreased SOCS3 levels comparable to the sh-NC empty vector (S1A-B). Similarly, the transfection of ov-TLR4 or sh-TLR4 exhibited increased or decreased expression of TLR4 (Fig. S1C-D). Furthermore, the co-immunoprecipitation assay showed SOCS3 directly bound to TLR4. HG treatment could increase TLR4 expression but decrease SOCS3 expression compared to the NG group. The IP assay showed that HG reduced the interaction between SOCS3 and TLR4 (Fig. S1E). All those results suggested that HG stimulation regulated the interaction of SOCS3 and TLR4 to contribute to diabetic injury.

### The expression of SOCS3 was downregulated, and TLR4 was upregulated in high glucose induced HK-2 cells

**Fig. 2:**
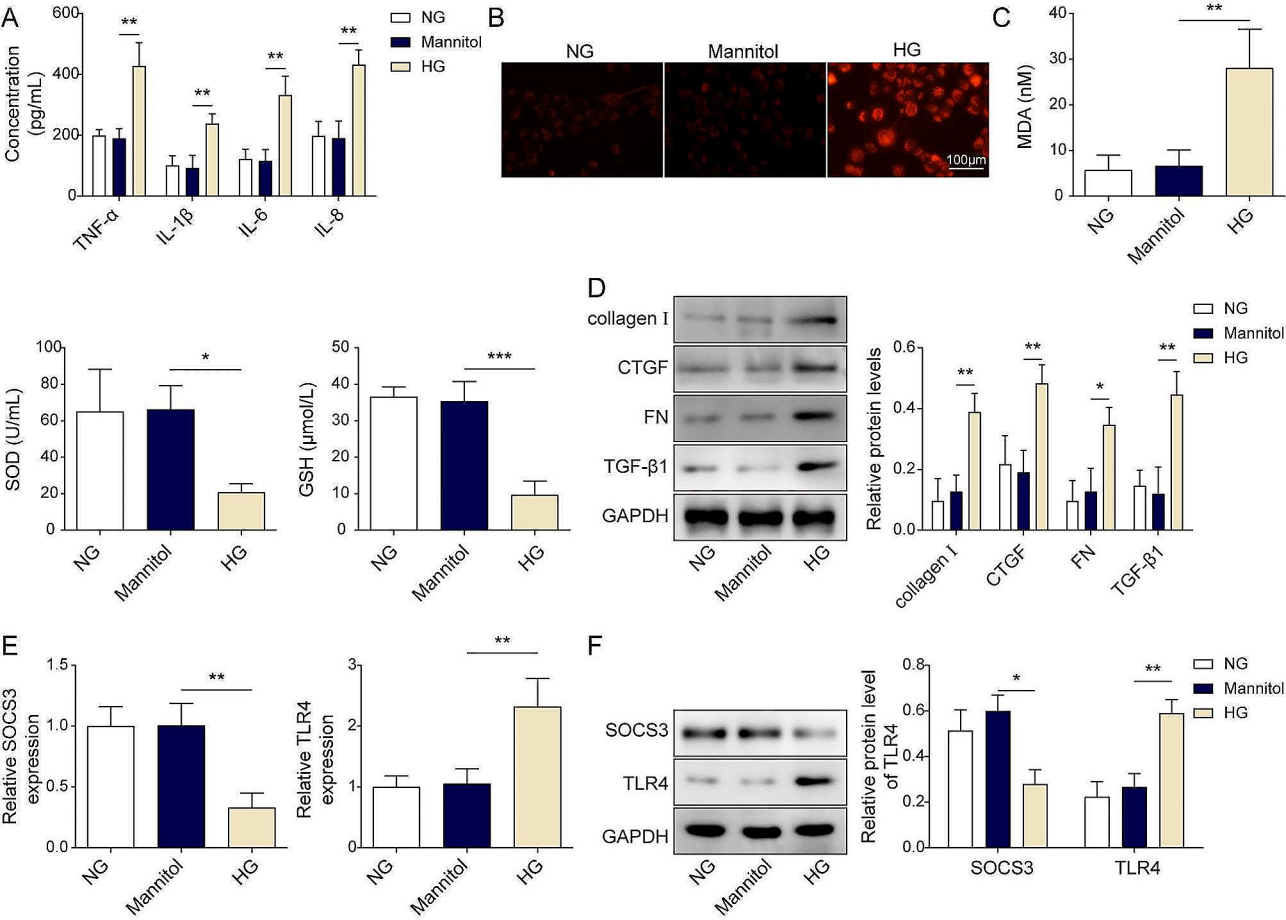
Effects of high glucose on the expressions of SOCS3 and TLR4 in HK-2 cells. (**A**). The secretions of cytokines were measured by ELISA assay. (**B**). ROS levels were detected by DCFH-DA fluorescence imaging at different times after mannitol or HG incubation. Scale bar = 100 μm. (**C**). The corresponding assay kit detected MDA, SOD, and GSH levels after mannitol or HG treatment. (**D**). The protein levels of collagen I, CTGF, FN, and TGF-β1 were detected by western blot assay after mannitol or HG treatment. (**E**-**F**). The mRNA and protein levels of SOCS3 and TLR4 were evaluated in HK-2 cells with mannitol or HG treatment by RT-qPCR and western blot, respectively. *P < 0.05, **P < 0.01, ***P < 0.001 versus the corresponding control. Data are representative of three independent experiments

Subsequent experiments by ELISA showed that high glucose (HG) stimulation led to remarkably increased cytokines, including TNF-α, IL-6, IL-1β, and IL-8 compared to NG and mannitol (Fig. 2A). Using the probe DCFDA, we found HG exposure induced a dramatical increase in ROS production (Fig. 2B) and MDA (Fig. 2C), while the decreased SOD and GSH, indicating that HG-induced oxidative stress. Under HG conditions, the expressions of collagen I, CTGF, FN, and TGF-β1 were significantly upregulated in HG-induced cells (Fig. 2D). Furthermore, HG treatment led to evident downregulation of SOCS3 and upregulation of TLR4 in mRNA and protein levels (Fig. 2E and F). However, compared with the HG group, we observed that mannitol exhibited no significant change in the secretion of ROS, MDA, SOS, GSH, and the expressions of collagen 1, CTGF, FN, TGF-β1, SOCS3, and TLR4. Besides, db/db mice showed increased blood glucose and insulin levels relative to db/m mice (Fig. S2A-B). H&E staining (Fig. S2C) in liver tissue sections showed swollen and increased cellular injuries in db/db mice. Furthermore, the db/db mice showed increased serumal ALT, AST, and ALP (Fig. S2D). Previous studies showed that circulating adiponectin and leptin serum levels are important for energy homeostasis and insulin sensitivity. Our further results showed that the circulating leptin was increased, but adiponectin was decreased in the diabetic mice (Fig. S2E-F). Compared to the db/db group, wogonin decreased the IPGTT, IPITT, liver structure damages, liver enzymes (ALP, AST, ALP) and leptin, but increased adiponectin (Fig. S2A-F). Those results suggested that SOCS3 and TLR4 probably attributed to HG-induced renal cell injuries by regulating the serumal inflammatory factors, liver energy homeostasis, and insulin sensitivity.

### Wogonin attenuated high glucose-induced injuries in HK-2 cells

**Fig. 3:**
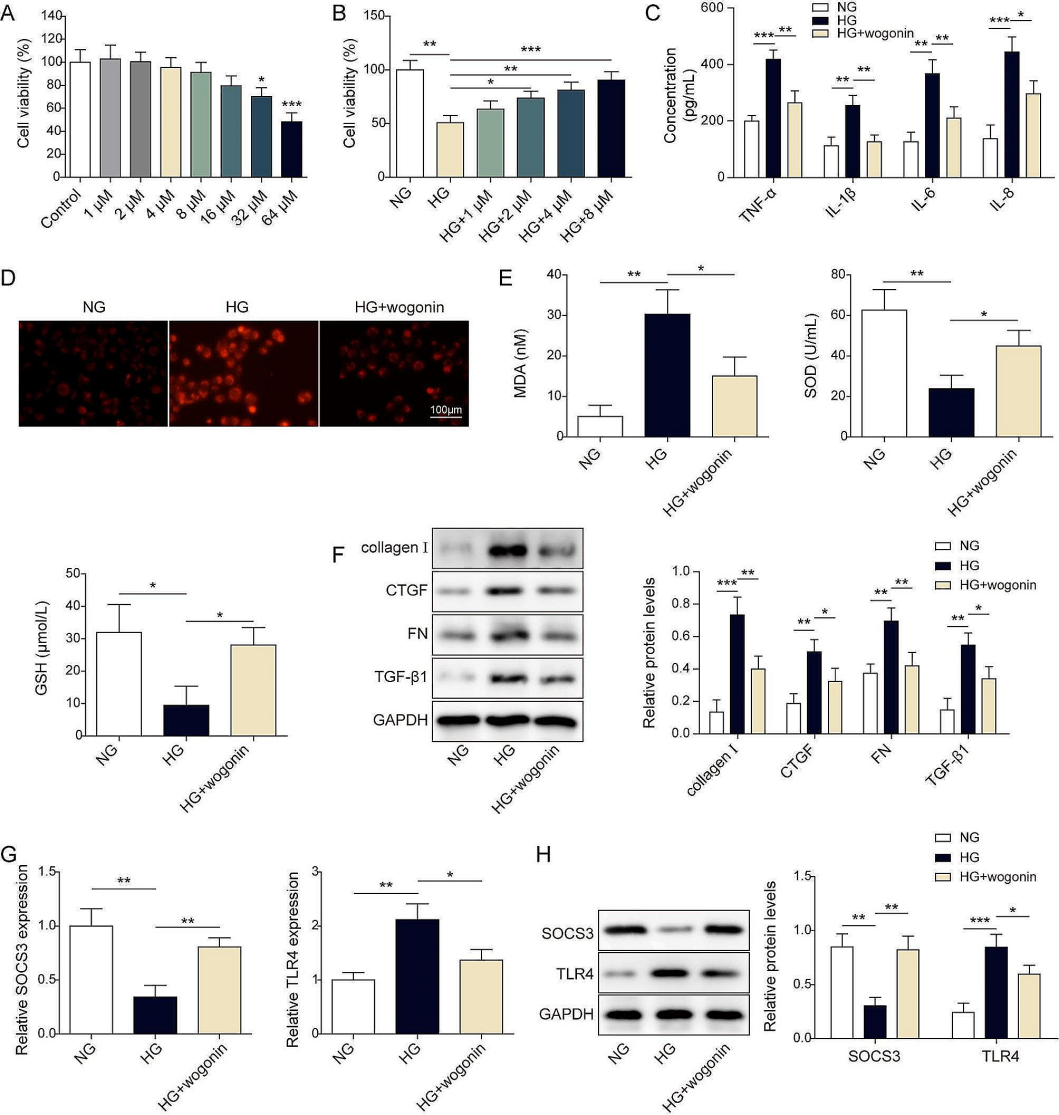
Wogonin attenuated high glucose-induced injuries in HK-2 cells. HK-2 cells were treated with vehicle, wogonin (1–64 µM) single or by treated with vehicle control, HG, or HG + wogonin (8 µM) for 24 h. Then, (**A**-**B**) cell survival was assessed by CCK-8 assay. (**C**) The cytokines levels were measured by ELISA. (**D**) ROS levels were detected by DCFH-DA. Scale bar = 100 μm. (**E**) MDA, SOD, and GSH were detected by the corresponding assay kit. (**F**) The protein levels of collagen I, CTGF, FN, and TGF-β1 were detected by western blot. (**G**) The mRNA levels of SOCS3 and TLR4 were detected by RT-qPCR. (**H**) The protein levels of SOCS3 and TLR4 were detected by western blot. *P < 0.05, **P < 0.01, ***P < 0.001 versus the corresponding control. Data are representative of three independent experiments

As shown in Fig. 3A, our data indicated that wogonin (< 16 µM) exhibited slight effects on cell viability, but high concentrations of wogonin (32–64 µM) led to a dramatic decrease in cell survival compared to the control group. We also found that wogonin exhibited a protective role in cell viability in a dose-dependent manner (1–8 µM). We chose a relatively optimal concentration (8 µM) in the following experiments (Fig. 3B). Subsequently, we treated HK-2 cells with the vehicle, HG, or HG + woginin for 24 h and detected their effects. Our results showed that HG led to significant cytokine secretion compared to the control group. However, wogonin diminished the HG-induced increase of cytokines (Fig. 3C). Additionally, wogonin antagonized HG-induced high levels of ROS (Fig. 3D) and MDA (Fig. 3E). In contrast, the anti-oxidative stress proteins SOD and GSH were robustly reversed by wogonin compared with HG-induced low level (Fig. 3E). Wogonin dramatically downregulated HG-induced high collagen I, CTGF, FN, and TGF-β1 (Fig. 3F). Moreover, we observed that HG led to an apparent decrease of SOCS3 and increase of TLR4 (Fig. 3G and H). However, wogonin markedly reversed the effects of HG on SOCS3 and TLR4. All those data revealed that wogonin protected HK-2 cells from the toxicity induced by HG exposure.

### SOCS3 overexpression alleviates high glucose-induced renal cell injury by inhibiting TLR4

**Fig. 4:**
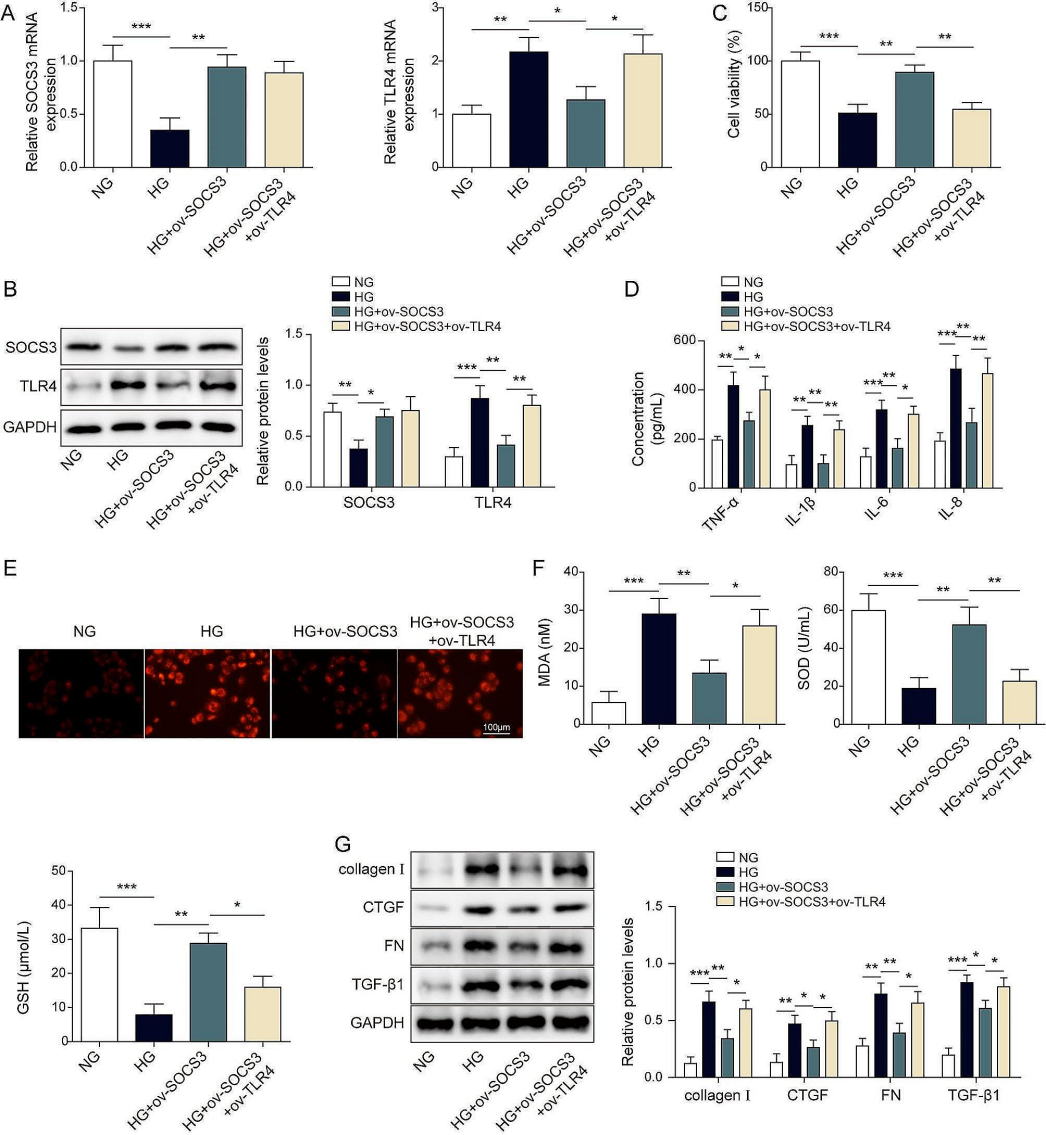
SOCS3 overexpression alleviates high glucose-induced renal cell injury by inhibiting TLR4 HK-2 cells were transfected with ov-SOCS3 single or together ov-TLR4 for 24 h, followed by 24 h treatment by vehicle or high glucose. (**A**-**B**) The mRNA and protein levels of SOCS3 and TLR4 were detected by RT-qPCR and western blot, respectively. (**C**) The cell viability was evaluated by CCK-8 assay. (**D**) The contents of cytokines were determined by ELISA assay. (**E**) A DCFH-DA Assay Kit detected the ROS levels. Scale bar = 100 μm. (**F**) The individual commercial kits measured the contents of MDA, SOD, and GSH. (**G**) The protein levels of collagen I, CTGF, FN, and TGF-β1 were measured by western blot. *P < 0.05, **P < 0.01, ***P < 0.001 versus the corresponding control. Data are representative of three independent experiments

To investigate whether SOCS3 overexpression influences HG-induced renal cell injury via regulating TLR4, we developed the constructs of ov-NC, ov-SOCS3, and ov-TLR4. Our results showed that ov-SOCS3 or ov-TLR4 transfection significantly increased the expression level of SOCS3 or TLR4 relative to ov-NC (Fig. S1A-D). Subsequently, we transfected ov-SOCS3 single or together with ov-TLR4, followed by vehicle or HG administration. HG downregulated SOCS3 expression but upregulated TLR4 expression. However, SOCS3 overexpression remarkably reversed HG-induced inhibition in SOCS3 and promotion in TLR4. Importantly, we found that TLR4 overexpression increased the level of TLR4 but exhibited no effect on SOCS3 expression (Fig. 4A and B). HG significantly decreased the cell viability of HK-2 cells by CCK-8 assay, but SOCS3 overexpression antagonized the effect of HG. However, TLR4 overexpression diminished the protective role of SOCS3 overexpression (Fig. 4C). Similarly, we found that HG dramatically increased TNF-α, IL-6, IL-1β, IL-8 (Fig. 4D), ROS (Fig. 4E) and MDA (Fig. 4F). However, HG decreased SOD and GSH (Fig. 4F). In contrast, SOCS3 overexpression strikingly inhibited the increased secretions of cytokines, ROS and MDA productions as well as the decreased levels of SOD and GSH by HG-treatment. Compared with SOCS3 overexpression, co-transfection of ov-SOCS3 and ov-TLR4 diminished the protective role of SOCS3 overexpression (Fig. 4D and F). Additionally, we found that HG caused high levels of renal fibrosis-related proteins (collagen I, CTGF, FN, and TGF-β1), but SOCS3 attenuated HG-induced toxic effects. However, TLR4 overexpression reversed the protective roles of SOCS3 (Fig. 4G). All those data revealed that SOCS3 overexpression alleviated cell injury via inhibiting TLR4 expression under HG conditions.

### Knockdown of TLR4 inhibited AIM2 inflammasome activation via the JAK/STAT to alleviate HG-induced HK-2 cell injury

**Fig. 5:**
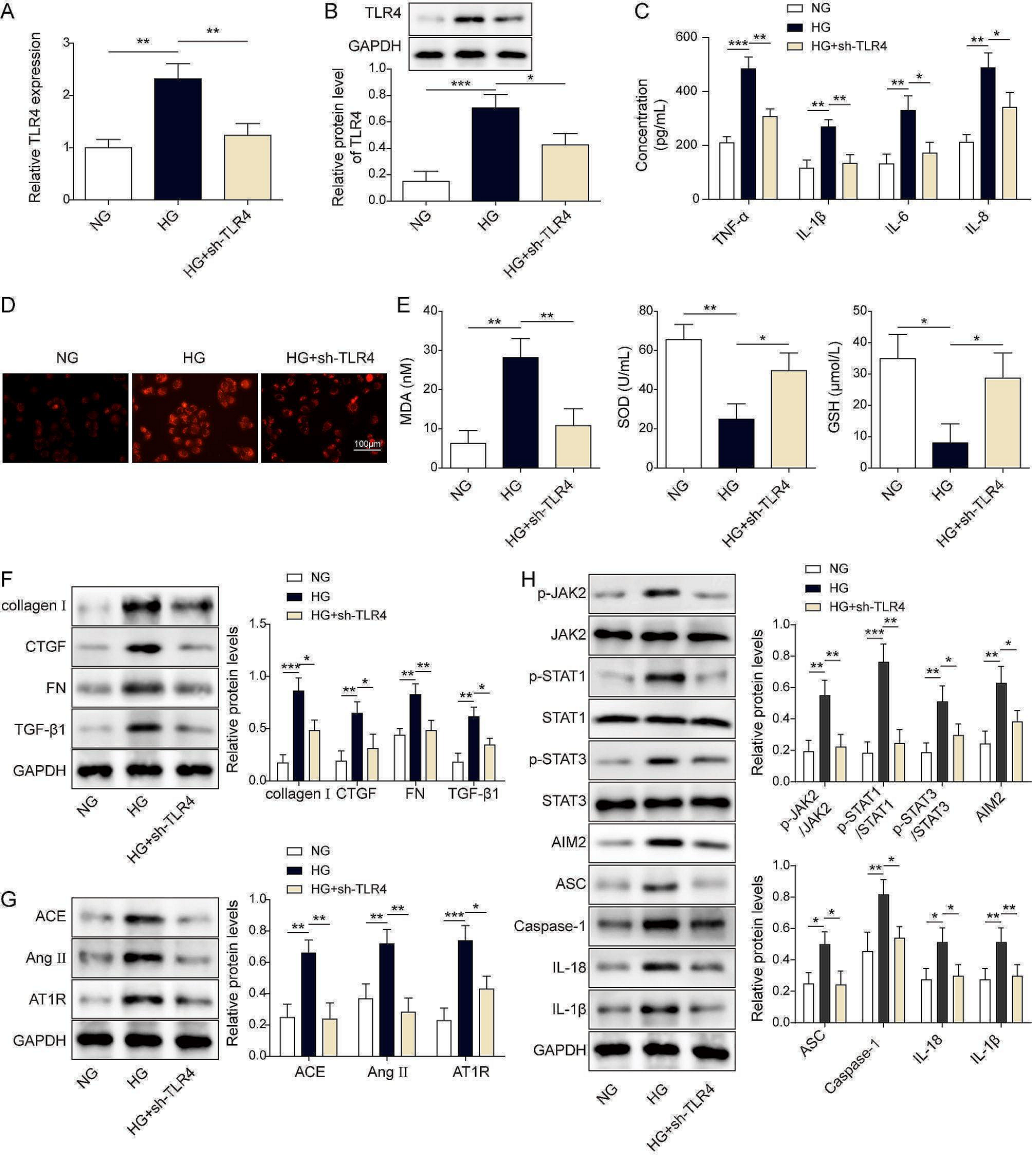
The knockdown of TLR4 inhibited AIM2 inflammasome activation via the JAK/STAT signaling pathway to alleviate high glucose exposure-induced HK-2 cell injury. HK-2 cells were transfected with shRNA empty vector or sh-TLR4, followed by exposure to high glucose. (**A**-**B**) The mRNA and protein levels of TLR4 were detected by RT-qPCR (**A**) and western blot assay (**B**). (**C**) The contents of cytokines were measured by ELISA assay. (**D**) The ROS levels were detected by DCFH-DA fluorescence imaging. Scale bar = 100 μm. (**E**) The individual commercial kits evaluated MDA, SOD, and GSH. (**F**) The protein levels of collagen I, CTGF, FN, and TGF-β1 were detected by western blot assay. (**G**) The protein levels of ACE, Ang II, and AT1R were detected by western blot assay. (**H**) The protein levels of p-JAK2, JAK2, p-STAT1, STAT1, p-STAT3, STAT3, AIM2, ASC, caspase-1, IL-18 and IL-1β were detected by western blot assay. *P < 0.05, **P < 0.01, ***P < 0.001 versus the corresponding control. Data are representative of three independent experiments

Firstly, we developed the constructs of sh-NC or sh-TLR4. Our data revealed that sh-TLR4 transfection decreased TLR4 levels relative to sh-NC transfection (Fig. S1C-D). Subsequently, we investigated the effect of TLR4 on HG-related inflammatory response. TLR4 knockdown significantly inhibited HG-induced high levels of TLR4 in HK-2 cells (Fig. 5A-B). Also, HG led to high levels of cytokines, ROS, and MDA and low levels of SOD and GSH, but after suppressing TLR4 by the transfection of sh-TLR4, the levels of cytokines, ROS, and MDA were evidently decreased, but the levels of anti-oxidative SOD and GSH were dramatically increased (Fig. 5C-E). Additional experiments showed that HG caused high levels of renal fibrosis-related proteins (collagen I, CTGF, FN, TGF-β1), renin-angiotensin system (RAS)(ACE, Ang II, and AT1R), JAK-STAT signaling and its inflammasome -related molecules (p-JAK2/JAK2, p-STAT1/STAT1, p-STAT3/STAT3, AIM2, ASC, caspase-1, IL-18 and IL-1β) were all significantly upregulated. However, knockdown of TLR4 markedly attenuated the effects of HG exposure (Fig. 5F-H). These findings revealed that TLR4 inhibition distinctly abolished JAK/STAT signaling-mediated inflammatory response by blocking the formation of AIM2 inflammasomes.

### Wogonin alleviated high glucose-induced HK-2 cell injury via inhibition of TLR4-regulated JAK/STAT pathway and AIM2 inflammasome activation through upregulation of SOCS3

**Fig. 6:**
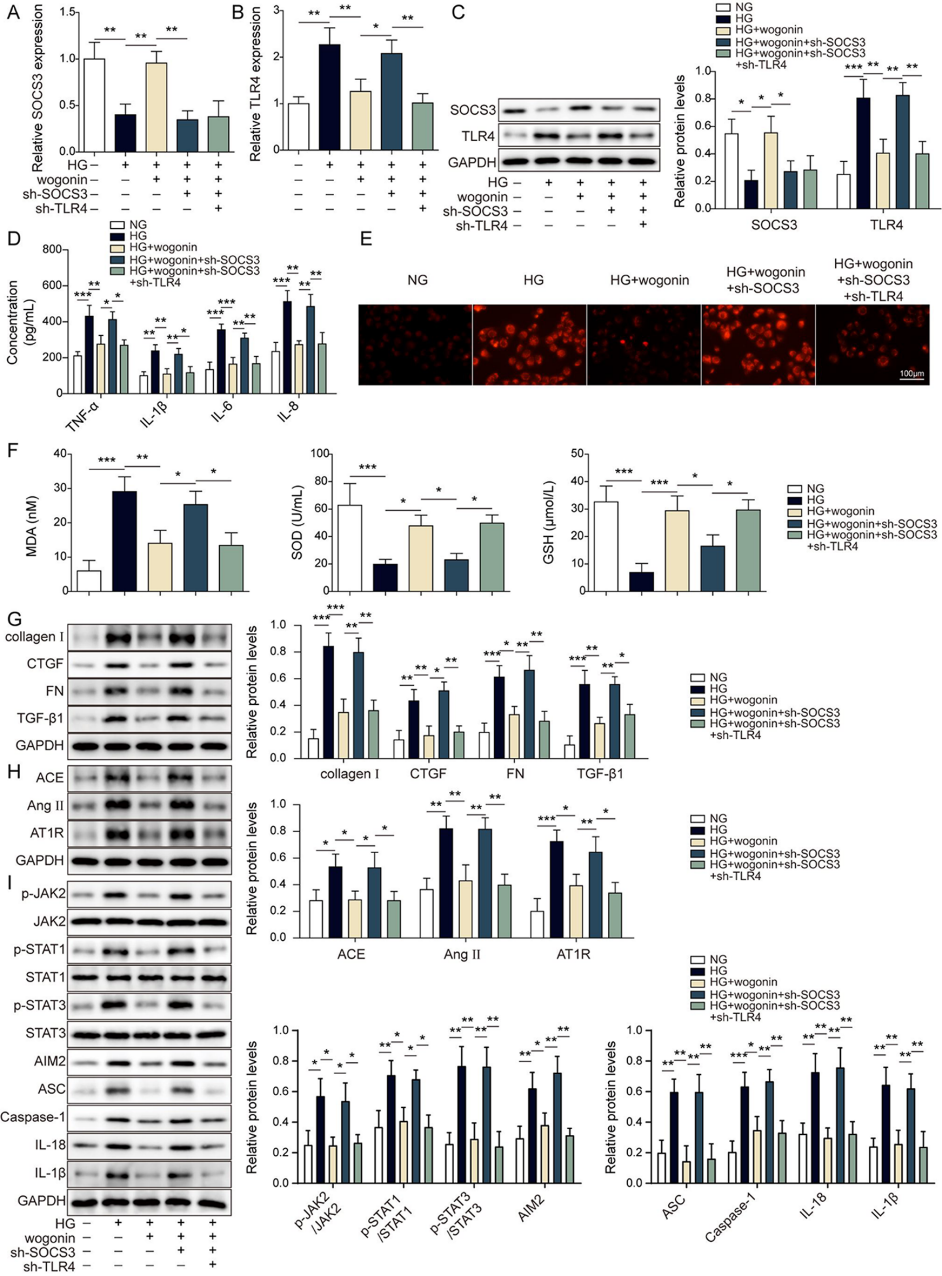
Wogonin alleviated high glucose-induced injury via inhibitionTLR4-regulatedated JAK/STAT pathway and AIM2 inflammasome activation through upregulation of SOCS3 HK-2 cells were individually transfected with shRNA empty vector, sh-SOCS3 single with sh-TLR4 for 24 h, followed by HG or HG + wogonin stimulation. (**A**-**B**) The mRNA levels of SOCS3 (**A**) and TLR4 (**B**) were detected by RT-qPCR. (**C**) The protein levels of SOCS3 and TLR4 were measured by western blot assay. (**D**) ELISA evaluated the contents of cytokines. (**E**) The ROS levels were assessed by DCFH-DA fluorescence imaging. Scale bar = 100 μm. (**F**) The respective commercial kits determined the contents of MDA, SOD, and GSH. (**G**) The protein levels of collagen I, CTGF, FN, and TGF-β1 were detected by western blot assay. (**H**) The protein levels of ACE, Ang II, and AT1R were detected by western blot assay. (**I**) The protein levels of p-JAK2, JAK2, p-STAT1, STAT1, p-STAT3, STAT3, AIM2, ASC, caspase-1, IL-18 and IL-1β were detected by western blot assay. *P < 0.05, **P < 0.01, ***P < 0.001 versus the corresponding control. Data are representative of three independent experiments

Further, we explored whether wogonin participated in HG-induced cytotoxicity via the SOCS3-mediated TLR4/JAK/STAT signaling pathway. Firstly, sh-SOCS3 or sh-TLR4 was transfected into HK-2 cells, and the results showed that sh-SOCS3 or sh-TLR4 significantly decreased the expression level of SOCS3 or TLR4 relative to sh-NC, respectively (Fig. S1A-D). Our results indicated that compared with only wogonin treatment, the knockdown of SOCS3 significantly antagonized wogonin-enhanced high SOCS3 under HG conditions (Fig. 6A-C). However, simultaneous inhibition of SOCS3 and TLR4 inhibited SOCS3 knockdown-induced increased TLR4 in HG and wogonin-co-treated HK-2 cells (Fig. 6A-C). These findings suggested that wogonin probably upregulated SOCS3 to diminish the expression of TLR4. Further experiments identified that wogonin decreased HG-induced high levels of ROS and MDA but increased HG-induced low levels of SOD and GSH (Fig. 6D-F). Compared with control, sh-SOCS3 transfection significantly reversed wogonin’s protective role in HG-induced high levels of cytokines. However, when both SOCS3 and TLR4 were silenced, the cytokines were decreased compared to that of only SOCS3 knockdown in HG and wogonin-cotreated cells, suggesting that wogonin exhibited the protective role through elevating SOCS3 to reduce TLR4-mediated inflammation (Fig. 6D-F). Furthermore, we also found that wogonin treatment significantly decreased the increased expressions of renal fibrosis-related proteins, renin-angiotensin system-related proteins, as well as JAK/STAT signaling molecules, and inflammasome protein AIM2, ASC, caspase-1, IL-18, IL-1β. However, when SOCS3 was silenced, the effects of wogonin were reversed. On the contrary, sh-TLR4 attenuated the enhanced impact of sh-SOCS3 (Fig. 6G-I). All those data indicated that wogonin protected HG-induced renal cell injuries through upregulating SOCS3 to inhibit TLR4/JAK/STAT signaling the pathway-mediated formation of AIM2 inflammasome.

### Wogonin inhibited TLR4 to ameliorate renal injury via upregulation of SOCS3 in db/db mice

**Fig. 7:**
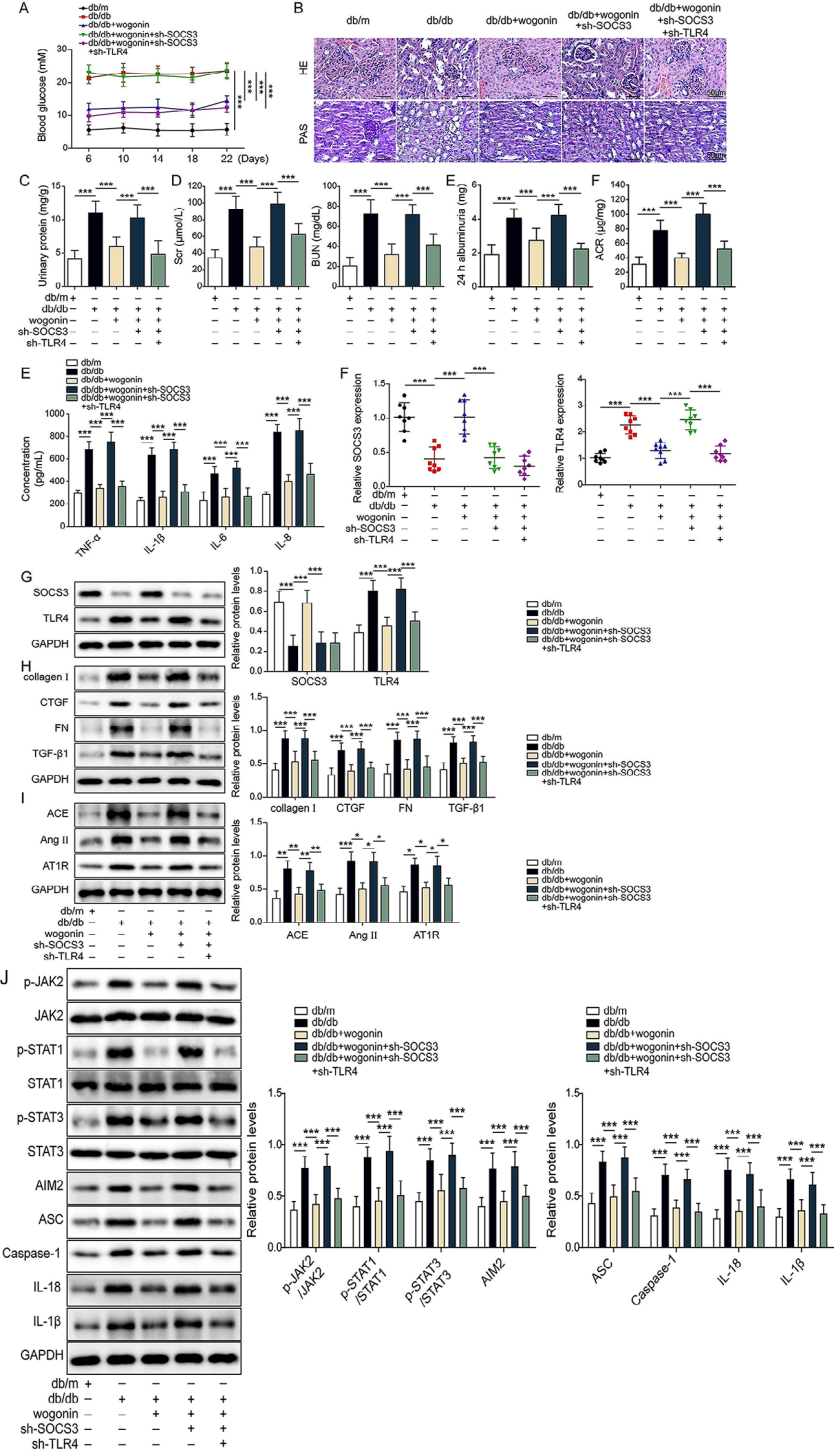
Wogonin inhibited TLR4 to ameliorate renal injury via upregulation of SOCS3 in db/db mice. db/db mice were injected with adenovirus sh-SOCS3 or sh-SOCS3 + sh-TLR4, followed by treatment by vehicle or wogonin. (**A**) Blood glucose levels were measured using an automated chemistry analyzer. (**B**) The pathological features and glucose in the renal tissues were evaluated by hematoxylin, eosin (HE), and periodic acid-Schiff base (PAS). Scale bar = 50 μm. (**C**-**D**) The urinary protein, serum creatinine values (Scr), and blood urea nitrogen (BUN) were evaluated by an automated chemistry analyzer. (**E**-**F**) 24-hour albumin and urinary albumin-to-creatinine ratio (ACR) were detected by ELISA assay in HG mice without or with wogonin, wogonin + sh-SOCS3, wogonin + sh-SOCS3 + sh-TLR4 (**E**) ELISA evaluated the levels of cytokines. (**F**-**G**) The mRNA and protein levels of SOCS3 and TLR4 were detected by RT-qPCR and western blot, respectively. (**H**) The protein levels of collagen I, CTGF, FN, and TGF-β1 were detected by western blot assay. (**I**) The protein levels of ACE, Ang II, and AT1R were detected by western blot assay. (**J**) The protein levels of p-JAK2, JAK2, p-STAT1, STAT1, p-STAT3, STAT3, AIM2, ASC, caspase-1, IL-18 and IL-1β were detected by western blot assay. *P < 0.05, **P < 0.01, ***P < 0.001 versus the corresponding control. Data are representative of three independent experiments

In the following, we developed db/m, db/db mice, and db/db mice with SOCS3 single or both SOCS3 and TLR4 knockdown by injection of lentivirus. The db/db mice exhibited higher blood glucose levels within the experimental duration (Days 6–22) than those of db/m mice. When mice were treated with wogonin, the blood glucose levels were significantly decreased. Knockdown of SOCS3 inversely promoted the high blood glucose level. However, when both SOCS3 and TLR4 were suppressed, the blood glucose levels were significantly attenuated compared with those of only the groups of SOCS3 knockdown after wogonin administration (Fig. 7A). Additional H&E and PAS staining assays showed that HG exposure induced renal tubular and interstitial fibrosis, mesangial matrix hyperplasia, and increased basement membrane thickness, but wogonin dramatically alleviated the pathological features. Knockdown of SOCS3 exacerbated HG-induced renal injury even if wogonin was administered. However, when SOCS3 and TLR4 were knocked down, wogonin exhibited strong protection compared with only SOCS3 knockdown (Fig. 7B). Similarly, we found that the db/db mice had higher levels of urinary protein, Src, BUN, and cytokines as well as urinary albumin and ACR compared with these of db/m mice. Wogonin dramatically decreased their levels compared with the db/db groups. Knockdown of SOCS3 did not rescue HG-induced high urinary protein, Src, BUN, cytokines, urinary albumin, and ACR after wogonin therapy. However, both knockdowns of SOCS3 and TLR4 recovered HG-induced high urinary protein, Src, and BUN, as well as urinary albumin and ACR after wogonin treatment (Fig. 7C-E). The db/db mice exhibited lower SOCS3 and higher TLR4 than db/m mice. After wogonin treatment, the db/db mice showed increased SOCS3 and decreased TLR4. However, when SOCS3 was knocked down, wogonin did not rescue the reduced SOCS3 and antagonized the increased TLR4. Knockdown of TLR4 with SOCS3 robustly decreased TLR4 but did not change the level of SOCS3 compared with only SOCS3 knockdown after wogonin treatment in db/db mice (Fig. 7F-G). As expected, HG led to high levels of fibrosis marker protein, RAS-related proteins, JAK/STAT signaling molecules, and inflammasome protein AIM2, caspase-1, IL-18, and IL-1β. After wogonin treatment, their levels significantly decreased. Only SOCS3 knockdown did not reduce HG-induced high levels of fibrosis marker proteins, RAS-related proteins, JAK/STAT signaling molecules, and inflammasome protein AIM2, caspase-1, IL-18 and IL-1β (Fig. 7H-J). In contrast, both SOCS3 and TLR4 knockdowns exhibited dramatically enhanced effects of wogonin by the decreased levels of fibrosis marker proteins, RAS-related proteins, JAK/STAT signaling molecules, and inflammasome proteins protein AIM2, caspase-1, IL-18, and IL-1β compared with those of only SOCS3 knockdown in db/db mice after wogonin therapy. All those results suggested that wogonin ameliorated HG-induced renal cell injuries in diabetic mice, possibly via upregulating SOCS3 to inhibit TLR4/JAK/STAT signaling pathway and its downstream AIM2 inflammasome formation.

## Discussions

Increasing evidence showed that diabetes mellitus (DM) is closely associated with inflammatory responses caused by hyperglycemia (Lontchi-Yimagou et al. 2013). Wogonin is a traditional Chinese herb. During the past several years, several studies pointed out that wogonin protects against diabetic diseases, such as diabetic cardiomyopathy (Khan et al. 2016) and diabetic nephropathy (Liu et al. 2022). This study found that HG significantly downregulated SOCS3 and upregulated TLR4, contributing to diabetic renal inflammation. Additional experiments identified that HG led to DN through the high levels of ROS, blood sera MDA, Src, and BUN, increased cytokine release, and renin-angiotensin system activation. Further experiments showed HG-induced cytotoxicity via inhibiting SOCS3/TLR4/JAK/STAT-mediated inflammasome activation. Importantly, we also confirmed that wogonin administration dramatically recovered the decreased level of SOCS3 by HG exposure, providing us with new evidence for protecting wogonin in DN.

During the past decade, SOCS3 acted as a negative modulator of inflammation to negatively regulate JAK/STAT pathway-related inflammation, which is a crucial step for developing insulin resistance and diabetic diseases (Haider et al. 2022). The previous study found that SOCS3 inhibition increased insulin sensitivity, indicating that SOCS3 might be a therapeutic target for diabetic diseases (Yang et al. 2010). The previous study showed that SOCS3 inhibition in diabetic patients exhibited a high level of system inflammation markers, such as C-reactive protein (CRP) and IL-1β (Haider et al. 2022). SOCS3 regulated inflammatory response, possibly through regulating p65 protein translocation from cytosol to nuclear and enhancing the activation of TLR4-dependent NLRP3/AIM2 inflammasome (Zhang et al. 2020a, b). A previous study showed that SOCS3 induced by wogonin regulates the PI3K-mediated MAPK signaling pathway (Nam et al. 2014). Our study found that HG exposure led to the downregulation of SOCS3, and wogonin treatment reversed HG-induced SOCS3 downregulation, which is consistent with the previous finding, suggesting that wogonin protects diabetic renal diseases by regulating SOCS3 expression.

Previous studies showed that TLR4 signaling is one of the major signaling pathways responsible for the pyroptosis of diabetic tubular cells (Wang et al. 2019). The microRNA miR-195 sponged and inhibited TLR4 to suppress the nuclear factor-κB (NF-κB) signaling pathway-dependent cytokine release (Zhu et al. 2021). Recently, several experiments showed that wogonin inhibited inflammatory response by suppressing TLR4-NF-κB-mediated inflammation (Wang et al. 2015; Xu et al. 2016). The present study observed that wogonin upregulated SOCS3 and downregulated TLR4, indicating that wogonin protects DN possibly through regulating the SOCS3/TLR4 axis to reduce HG-induced cytotoxicity. Thus, the SOCS3/TLR4 axis might be a new therapeutic target for DN.

Diabetic nephropathy is a multifactorial disease. Accumulating evidence pointed out that the development of renal changes in diabetes experienced five stages, from early hyperfunction and hypertrophy to end-stage renal failure with diabetic nephropathy-induced uremia(Mogensen et al. 1983). Previous studies have shown that hyperglycemia is the primary force for the progression of DN. Among many pathways, the inflammation pathway is central to DN (Wada and Makino 2013). Previous experiments indicated that HG-induced TLR4 expression upregulated IL-6 and chemokine ligand 2, promoting tubulointerstitial inflammatory response (Lin et al. 2012). Growing evidence showed that several pathways such as Janus kinase/signal transducers and activators of transcription (JAK/STAT), nuclear transcription factor kappa B (NF-κB), and nuclear factor erythroid 2-related factor 2 (Nrf2) are responsible for the inflammatory activation. Among those pathways, the hyperglycemia-activated JAK/STAT pathway plays a critical role in renal damage by participating in several inflammatory processes, such as angiotensin II-dependent hypertrophy of mesangial cells, and the synthesis of fibronectin, collagen IV and TGF (Lin et al. 2012). This study exhibited that wogonin alleviated diabetic neuropathy by the attentuation of renal inflammation, tubular epithelial structural injury and fibrosis. Importantly, we revealed wogonin protected diabetic neuropathy via modulating through regulating JAK/STAT/AIM2, which is distincted from PI3K/Akt/NF-κB (Lei et al. 2021) and NF-κB and TGF-β1/Smad3 (Zheng et al. 2020) signaling pathways.

**Fig. 8:**
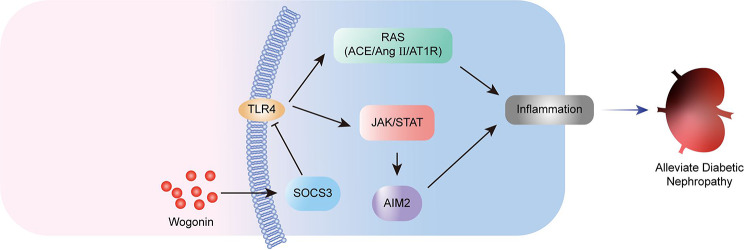
Schematic diagram for wogonin-suppressing inflammation and alleviating diabetic neuphropathy

Furthermore, the HG-activated JAK/STAT pathway was closely associated with renal fibrosis in humans and mice with diabetic renal diseases (Brosius 2008). In the present study, our results revealed that HG significantly upregulated TLR4, JAK/STAT, RAS, and renal fibrosis-related molecules, consistent with previous reports. Interestingly, our finding confirmed that wogonin dramatically upregulated SOCS3, which antagonized HG-induced downregulation of SOCS3. Meanwhile, the upregulation of SOCS3 by wogonin significantly suppressed TLR4 expression, blocked its downstream JAK/STAT pathway, and alleviated HG-induced inflammatory response and activation of RAS, which suggested that wogonin protected HG-induced renal cytotoxicity by upregulating SOCS3 and suppressing TLR4/JAK/STAT pathway (Fig. 8). Although several studies mentioned that wogonin exhibited a protective role in diabetic nephropathy, there are still few reports about whether wogonin has a therapeutic effect on diabetes patients.

## Conclusion

Our findings showed that wogonin exhibited the preventive role in HG-induced inflammatory response via inhibiting the TLR4-activated JAK/STAT pathway to inhibit AIM2 inflammasome activation. The further experiment confirmed that wogonin mediated inflammatory response and renal fibrosis by directly upregulating SOCS3 to decrease TLR4 expression, suggesting that SOCS3/TLR4 axis was a potential therapeutic target in DN. Wogonin might be a candidate drug to treat DN.

### Electronic supplementary material

Below is the link to the electronic supplementary material.

Supplementary Material 1

Supplementary Material 2

## Data Availability

All data collected and analyzed during the current study are available from the corresponding author on reasonable request.

## Abbreviations

SOCS3: Suppressor of cytokine signaling 3
DN: Diabetic nephropathy
TLR4: Toll-like receptor 4
JAK: Janus kinase
STAT: Signal transducer and activator of transcription
AIM2: Melanoma 2
HG: High glucose
Scr: Serum creatinine values
BUN: Blood urea nitrogen
SOD: Superoxide dismutase
GSH: Glutathione
ROS: Reactive oxygen species
RAS: Renin-angiotensin system
H&E: Hematoxylin, and eosin
PAS: Periodic acid-Schiff
ACR: Albumin to creatinine ratio
IPGTT: Intraperitoneal insulin tolerance test
IPITT: Intraperitoneal glucose tolerance test
ALP: Alkaline phosphatase
ALT: Alanine transaminase
AST: Aspartate aminotransferase
