# Supplementary figures and images for: Wogonin upregulates SOCS3 to alleviate the injury in Diabetic Nephropathy by inhibiting TLR4-mediated JAK/STAT/AIM2 signaling pathway
Source: Mol Med. 2024 Jun 6;30:78. doi: 10.1186/s10020-024-00845-4 (PMC11155057; doi:10.1186/s10020-024-00845-4)

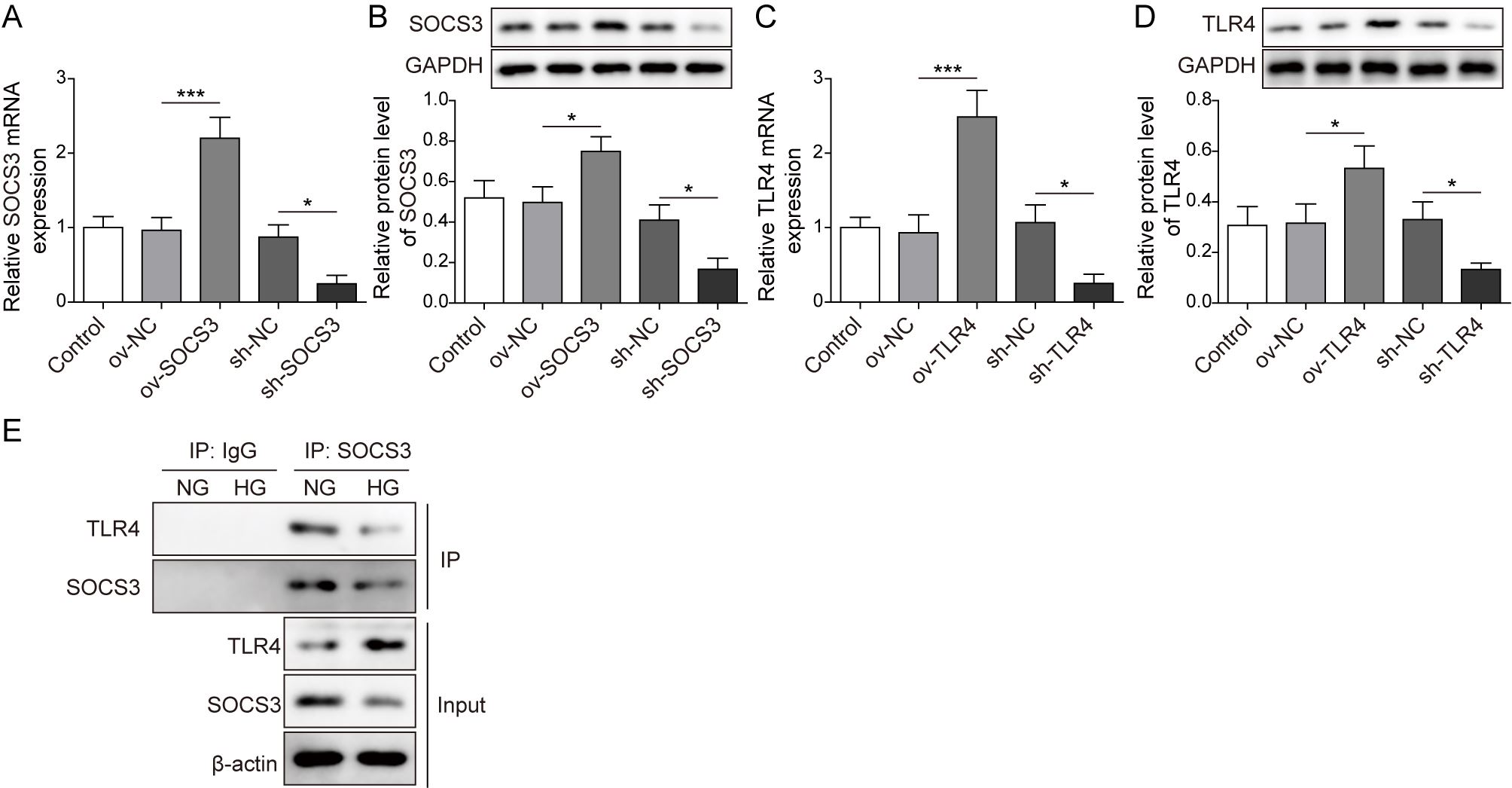

Supplement: Supplementary file 1 — Supplementary Material 1 [file 10020_2024_845_MOESM1_ESM.jpg]

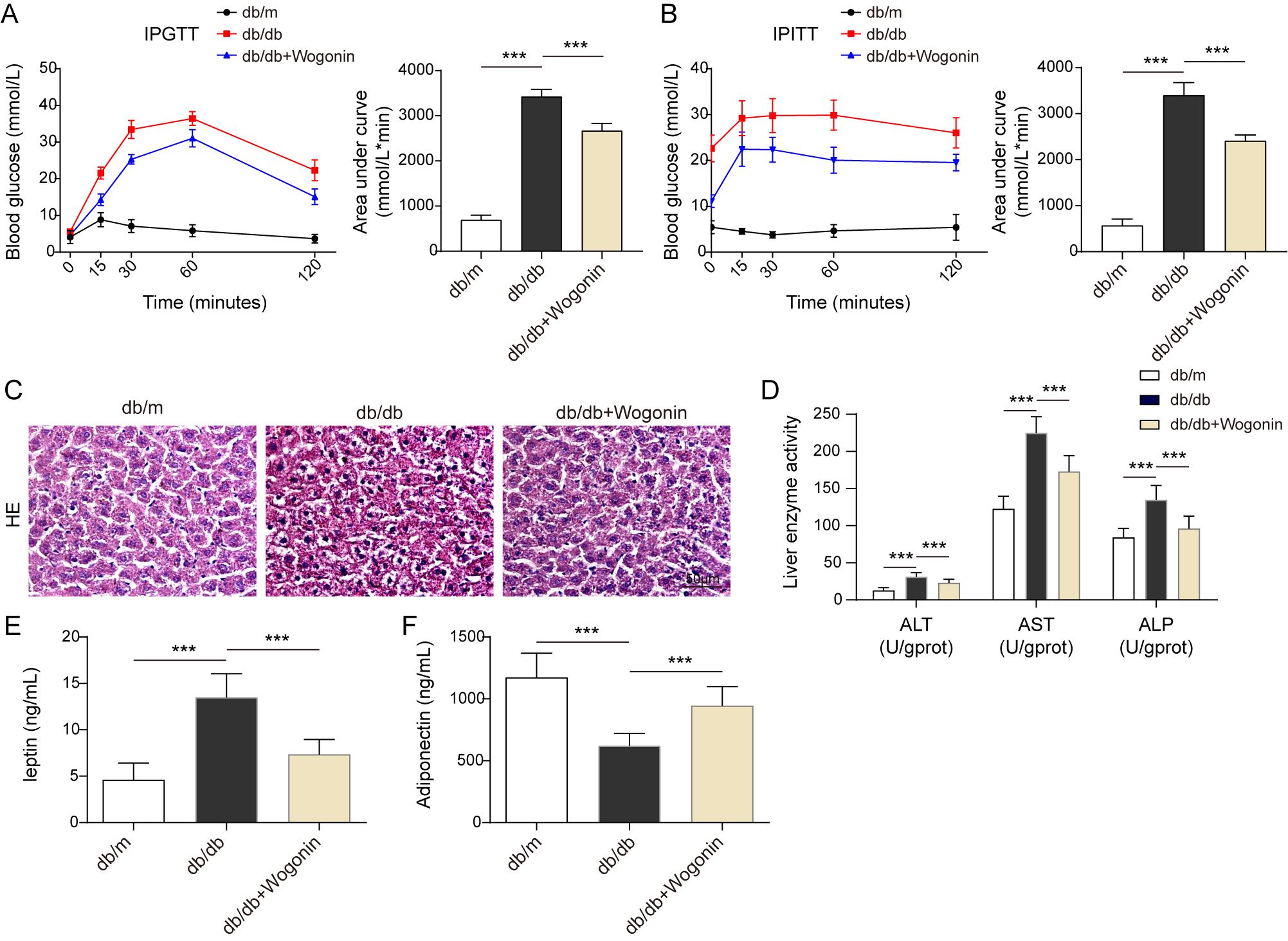

Supplement: Supplementary file 2 — Supplementary Material 2 [file 10020_2024_845_MOESM2_ESM.jpg]
